# COVID-19 Vaccine Uptake among US Adults According to Standard Occupational Groups

**DOI:** 10.3390/vaccines10071000

**Published:** 2022-06-23

**Authors:** Itunu Sokale, Juan Alvarez, Omar Rosales, Eric Bakota, Christopher I. Amos, Hoda Badr, Abiodun O. Oluyomi

**Affiliations:** 1 Department of Medicine, Epidemiology and Population Sciences Section, Baylor College of Medicine, Houston, TX 77030, USA; itunu.sokale@bcm.edu (I.S.); juan.alvarez@bcm.edu (J.A.); omar.rosales@bcm.edu (O.R.); chris.amos@bcm.edu (C.I.A.); hoda.badr@bcm.edu (H.B.); 2 Office of Science, Surveillance, and Technology, Harris County Public Health, Houston, TX 77030, USA; eric.bakota@phs.hctx.net; 3 Institute of Clinical and Translational Research, Baylor College of Medicine, Houston, TX 77030, USA

**Keywords:** COVID-19 vaccine, occupation, workplace, neighborhood, census tract

## Abstract

This cross-sectional ecological study examined the relationship between neighborhood-level standard occupational groups in the USA and COVID-19 vaccine uptake using 774 census tract data, each consisting of approximately 1600 housing units. The neighborhood-level COVID-19 vaccination uptake data were retrieved from Harris County Public Health, Harris County, Texas. The standard occupational group data were from the US Census Bureau. We calculated the incidence rate ratios (IRRs) for vaccine uptake using bivariate and multivariable Poisson regression models. In the adjusted models, we found that the healthcare practitioner/technician (IRR: 1.008; 95% CI: 1.003–1.014; *p* = 0.001), business/management/legal (IRR: 1.011; 95% CI: 1.008–1.013; *p* < 0.001), computer/engineering/life/physical/social science (IRR: 1.018; 95% CI: 1.013–1.023; *p* < 0.001), and arts/design/entertainment/sports/media (IRR: 1.031; 95% CI: 1.018–1.044; *p* < 0.001) occupational groups were more likely to have received the full regimen of a COVID-19 vaccine. On the contrary, the building/installation/maintenance/repair (IRR: 0.991; 95% CI: 0.987–0.995; *p* < 0.001), construction/extraction/production (IRR: 0.991; 95% CI: 0.988–0.995; *p* < 0.001), transportation/material moving (IRR: 0.992; 95% CI: 0.987–0.997; *p* = 0.002), food preparation/serving related (IRR: 0.995; 95% CI: 0.990–0.999; *p* = 0.023), and personal care/services (IRR: 0.991; 95% CI: 0.985–0.998; *p* = 0.017) groups were less likely to have received the complete dose of a COVID-19 vaccine. White-collar workers were more likely to be vaccinated than blue-collar workers. We adjusted for age, sex, and race/ethnicity in the multivariable analysis. The low vaccine uptake among certain occupational groups remains a barrier to pandemic control. Engaging labor-centered stakeholders in the development of vaccination interventions may increase uptake.

## 1. Introduction

The benefits of vaccination within public health interventions are widely recognized. Annually, immunizations prevent more than 2 million deaths from multiple preventable diseases [1]. Unfortunately, despite the current availability of safe and effective COVID-19 vaccines, the daily numbers of new cases and preventable deaths from COVID-19 in the United States (US) remain high. For example, in October 2021, the US average daily estimates for new COVID-19 cases and COVID-19-related deaths were 95,000 and 1400, respectively [2]. As a result, the US government and many non-governmental organizations have been making significant efforts to improve COVID-19 vaccine uptake among US workers across different occupational groups. This push is mainly because the low vaccine uptake puts individuals, coworkers, and clients at risk [3] and may prolong the pandemic.

Simultaneously, there is a growing body of literature on COVID-19 vaccination hesitancy, intention, and acceptability [3,4,5,6,7]. Vaccine hesitancy has been attributed primarily to concerns about vaccine safety and a mistrust of medical science [3,4]. While investigations into intention, acceptability, and hesitancy to participate in vaccination programs may serve as good predictors for vaccination behavior, the results could be too subjective and inadequate for elucidating actual vaccine uptake. Recently, healthcare workers across the globe have been in the spotlight, given their important role as frontline workers [4,5,8,9,10]. The findings of a state-wide study on nursing home and assisted living workers before the availability of the COVID-19 vaccines suggested that less than half of the workers were willing to participate in a vaccination program when one became available and that half of those uncertain workers would consider getting a COVID-19 vaccination in the future [5]. Additionally, a longitudinal study examined COVID-19 acceptability and uptake among frontline healthcare workers in California, USA. The results showed a progressive and significant increase in acceptability and vaccine uptake compared to the baseline data [4].

Although there has been a substantial focus on healthcare workers globally, including in the USA, there is limited information on the COVID-19 vaccination behavior among other occupational groups, specifically in the US. Nevertheless, a few recent works have addressed the influence of occupational groups on COVID-19 vaccine uptake in the US and other countries [11,12]. Occupation-based interventions have demonstrated efficacy in health promotion interventions, including tobacco control programs and influenza vaccine interventions [13,14]. Thus, occupation-based interventions may provide unique opportunities to strengthen COVID-19 vaccination uptake and help to control the pandemic. However, studies that have investigated the actual COVID-19 vaccination uptake among multiple occupational groups in the USA are scarce. In the current analysis, we assessed the associations between the neighborhood compositions of standard US occupational groups and neighborhood-level vaccine uptake in Harris County, Texas.

## 2. Materials and Methods

This cross-sectional ecological study used neighborhood-level data for the COVID-19 vaccine uptake between 14 December 2020 and 10 August 2021 in Harris County, Texas. The estimated 2020 population of Harris County was 4.72 million people [15], making it the third most populous county in the USA. The county has a very diverse population and a high number of foreign-born residents (26% of the county population) [15]. We received the data on the COVID-19 vaccination uptake from the Office of Science, Surveillance, and Technology (OSST), Harris County Public Health, Harris County, TX, USA. The OSST provided the number of residents in each Harris County census tract that had received the full regimen of a COVID-19 vaccination, as of 10 August 2021.

A census tract (CT) is a small, homogenous, and relatively permanent statistical subdivision of a county, which usually consists of 4000 residents or 1600 housing units [16]. A CT is often used to represent neighborhood-level factors in health studies on the US population. Of the 786 CTs in Harris County, the OSST provided data for 782, of which 774 had occupational data; hence, 774 CTs were used for this analysis. We retrieved the occupational group data (explanatory variables) from the US Census Bureau’s 2014–2018 American Community Survey (ACS) 5-year estimates dataset [17]. A total of 22 occupational groups, as classified by the US Bureau of Labor Statistics, were reorganized into 15 groups (Table 1). The occupational groups were analyzed individually and the percentage of CT residents that were employed in each occupational group was entered into the analysis workflows as a continuous variable. A multivariable analysis was run for each occupational group by adjusting for % Hispanic, % Black or African American, % female, and % under 40 years old (covariates). The covariates were retrieved from the US Census 2015–2019 ACS.

For our statistical analysis, we used a Poisson-based modeling protocol. To address the over-dispersion that was observed in the vaccine uptake dataset, we utilized a negative binomial regression (NBR) technique [18]. Given the unequal CT populations, the natural logarithm of the population was used as an offset variable in all NBR models. For each CT, we used the estimated population of CT residents who were at least 15 years old. The was due to the fact that the US health authorities were yet to officially approve vaccines for children under 15 years of age at the time that our vaccine uptake data were compiled. The effect estimates were expressed as relative risk (incidence rate ratios, IRRs) by exponentiating the Poisson regression coefficient. The explanatory variables were presented as counts (%). For interpretation, the expected number of COVID-19 vaccine uptake within a CT increases or decreases by a factor of the effect measure (IRR) when the population percentage in an occupation increases by 1 unit. Due to the non-independent nature of the occupational groups inside a specific CT, our models did not evaluate the relative effects on each occupational group vis-à-vis the other groups. Instead, each occupational group was entered into a pair of Poisson models. In the first model, vaccine uptake was regressed on just the occupational group alone (bivariate analysis) while in the second model, vaccine uptake was regressed on the occupational group alongside four other independent variables (% Hispanic, % Black or African American, % female, and % less than 40 years old; i.e., the adjusted covariates).

## 3. Results

Table 2 presents the associations between vaccine uptake and the standard US occupational groups at the census tracts level. In the bivariate analysis, a high vaccine uptake was associated with healthcare practitioner/technician, business/management/legal, computer/engineering/life/physical/social science, arts/design/entertainment/sports/media, sales/sales related, and education/training/library workers. In contrast, we observed a low vaccine uptake among social service/protective services, healthcare support, office/administrative support, farming/fishing/forestry, building/installation/maintenance/repair, construction/extraction/production, transportation/material moving, food preparation/serving related, and personal care/services workers.

After adjusting for age, race/ethnicity, and sex, people who were in the healthcare practitioner/technician (IRR: 1.008; 95% CI: 1.003–1.014; *p* = 0.001), business/management/legal (IRR: 1.011; 95% CI: 1.008–1.013; *p* < 0.001), computer/engineering/life/physical/social science (IRR: 1.018; 95% CI: 1.013–1.023; *p* < 0.001), and arts/design/entertainment/sports/media (IRR: 1.031; 95% CI: 1.018–1.044; *p* < 0.001) occupational groups were more likely to have received a COVID-19 vaccine. On the contrary, building/installation/maintenance/repair (IRR: 0.991; 95% CI: 0.987–0.995; *p* < 0.001), construction/extraction/production (IRR: 0.991; 95% CI: 0.988–0.995; *p* < 0.001), transportation/material moving (IRR: 0.992; 95% CI: 0.987–0.997; *p* = 0.002), food preparation/serving related (IRR: 0.995; 95% CI: 0.990–0.999; *p* = 0.023), and personal care/services (IRR: 0.991; 95% CI: 0.985–0.998; *p* = 0.017) workers were less likely to have received a COVID-19 vaccine after adjusting for age, sex, and race/ethnicity (See Supplemental Materials, Table S1, for the covariates results).

## 4. Discussion

Workplace interventions present a valuable opportunity to increase COVID-19 vaccine uptake and further minimize the impacts of the COVID-19 pandemic. Our results suggested a discrepancy in COVID-19 vaccine uptake between white-collar jobs versus blue-collar jobs. Similar to our findings, a study on national COVID-19 vaccine coverage according to occupation in England observed higher vaccination uptake among highly skilled professionals and lower vaccination rates among blue-collar workers [11]. In addition, a US social media-based study examined self-reported COVID-19 vaccine uptake among the major occupational groups [12]. The researchers found that the highest and lowest vaccination rates were among college teachers and manual workers, respectively. However, the authors could not exclude the possibility of survey duplication within the sample.

As mentioned previously, vaccine hesitancy studies are more prevalent than COVID-19 vaccine uptake studies. Even so, the vaccine hesitancy or attitude studies involving occupational groups have generally reported findings that were similar to ours. A US study on COVID-19 vaccine hesitancy according to occupation and employment reported variations in vaccine hesitancy among the occupational groups, with the highest vaccine hesitancy observed among manual workers [19]. An Italian study on attitudes toward potential COVID-19 vaccines and influenza vaccines reported lower intentions to receive a potential COVID-19 vaccine among blue-collar workers and farmers. In contrast, managers, white-collar workers, and teachers were willing to accept a COVID-19 vaccine when one became available [20].

The disparity in the vaccine uptake that we observed could have resulted from the higher educational attainment among professional workers, resulting in a better understanding of the risks of COVID-19 infection, the benefits of vaccinations, and greater access to preventive measures, including vaccination services. Studies have shown that people with highly paying jobs are more likely to enjoy better health than those with low-paying jobs and that an increase in the years of education increases the impact of education on health [21]. This study highlighted the disparity in COVID-19 vaccine uptake between white-collar and blue-collar workers. The workplace is an ecosystem comprising interdependent actors who interact to achieve individual and collective goals [22]. A workplace becomes unsafe when disparities in vaccination uptake across job levels are not addressed. For example, an institution with high vaccination rates among the management-level staff but low vaccination rates among the maintenance staff may face challenges in controlling COVID-19 infection.

Our results indicated that highly skilled professionals were more likely to be vaccinated than lay workers and these results remained significant after adjusting for age, sex, and race/ethnicity. Another study investigated COVID-19 vaccine hesitancy and uptake according to race and ethnicity in the USA and the United Kingdom (UK) [23]. In both countries, the authors found significantly lower rates of vaccine hesitancy among participants of a racial minority, while this only persisted in vaccine uptake among US respondents [23]. Another study examined gender differences in COVID-19 vaccine acceptance among working adults in Japan [24]. Their results indicated that older men were more likely to be willing to receive a vaccine. Similarly, another US study on health care workers reported higher acceptance of vaccination among respondents who were male, older, and White or Asian [25].

We acknowledge that our study had strengths and limitations. We analyzed the associations between COVID-19 vaccine uptake and occupational groups using census tract data from actual vaccination records, unlike previous studies that have examined attitudes, acceptance or hesitancy at an individual level. Our data could also inform preparedness strategies for future pandemics in terms of work-based interventions. Our analysis used area-level vaccine uptake data and a cumulative count from January 2021 to August 2021. Therefore, we could not rule out the possibility of ecological fallacy. In addition, the variations in COVID-19 vaccine uptake among occupational groups might have since improved due to the additional public health interventions to reduce morbidity and mortality from the disease as a result of more transmissible variants, including the Delta and Omicron variants. Future studies analyzing more recent data should describe the temporal changes in vaccine uptake among the occupational groups.

## 5. Conclusions

Given the variations in vaccine uptake among the occupational groups, there is a need to involve labor-centered organizations/stakeholders in developing COVID-19 vaccination interventions. Additionally, we recommend the development of workplace-based and union-level interventions for both institution-based and self-employed workers. Interventions could use multiple evidence-based and theory-grounded strategies to address vaccine mistrust, the risks of COVID-19, the benefits of vaccination, and access to vaccinations, including the provision of vaccination clinics to encourage vaccination among workgroups with low uptake rates.

## Figures and Tables

**Table 1:** The US Bureau of Labor Statistics classification of the standard occupational groups and our reclassification for the statistical analysis.

Original US Standard Occupational Groups		Reclassification for Statistical Analysis	
1	Community/Social Service	1	Social Service/Protective Services
2	Protective Service		
3	Healthcare Practitioner/Technician	2	Healthcare Practitioner/Technician
4	Healthcare Support	3	Healthcare Support
5	Management	4	Business/Management/Legal
6	Business/Financial		
7	Legal		
8	Computer/Mathematical	5	Computer/Engineering/Life/Physical/Social Science
9	Architecture/Engineering		
10	Life/Physical/Social Science		
11	Arts/Design/Entertainment/Sports/Media	6	Arts/Design/Entertainment/Sports/Media
12	Office/Administrative Support	7	Office/Administrative Support
13	Farming/Fishing/Forestry	8	Farming/Fishing/Forestry
14	Building/Grounds Cleaning/Maintenance	9	Building/Installation/Maintenance/Repair
15	Installation/Maintenance/Repair		
16	Construction/Extraction	10	Construction/Extraction/Production
17	Production		
18	Transportation/Material Moving	11	Transportation/Material Moving
19	Food Preparation/Serving Related	12	Food Preparation/Serving Related
20	Personal Care/Services	13	Personal Care/Service
21	Sales/Sales Related	14	Sales/Sales Related
22	Education/Training/Library	15	Education/Training/Library

**Table 2:** The associations between COVID-19 vaccine uptake in Harris County and the percentage of census tract residents that were employed in each of the US standard occupational groups (*n* = 774 census tracts) up to August 2021.

Occupational Groups	Bivariate Analysis ^a^			Multivariable Analysis ^a^		
	IRR	95% CI	*p*-Value	IRR ^b^	95% CI	*p*-Value
Social Service/Protective Services	0.99	0.97–0.99	< 0.001	0.996	0.988–1.005	0.399
Healthcare Practitioner/Technician	1.03	1.02–1.03	< 0.001	1.008	1.003–1.014	**0.001**
Healthcare Support	0.96	0.96–0.97	< 0.001	0.998	0.989–1.007	0.693
Business/Management/Legal	1.02	1.01–1.02	< 0.001	1.011	1.008–1.013	**< 0.001**
Computer/Engineering/Life/Physical/Social Science	1.03	1.02–1.03	< 0.001	1.018	1.013–1.023	**< 0.001**
Arts/Design/Entertainment/Sports/Media	1.08	1.06–1.09	< 0.001	1.031	1.018–1.044	**< 0.001**
Office/Administrative Support	0.99	0.98–0.99	< 0.001	0.997	0.993–1.001	0.089
Farming/Fishing/Forestry	0.93	0.90–0.96	< 0.001	0.989	0.958–1.021	0.485
Building/Installation/Maintenance/Repair	0.98	0.97–0.98	< 0.001	0.991	0.987–0.995	**< 0.001**
Construction/Extraction/Production	0.99	0.98–0.99	< 0.001	0.991	0.988–0.995	**< 0.001**
Transportation/Material Moving	0.97	0.97–0.98	< 0.001	0.992	0.987–0.997	**0.002**
Food Preparation/Serving Related	0.98	0.97–0.98	< 0.001	0.995	0.990–0.999	**0.023**
Personal Care/Services	0.98	0.97–0.98	< 0.001	0.991	0.985–0.998	**0.017**
Sales/Sales Related	1.01	1.01–0.02	< 0.001	0.999	0.994–1.004	0.658
Education/Training/Library	1.03	1.02–1.04	< 0.001	1.002	0.995–1.008	0.628

^a^ Each occupational group was entered into a pair of Poisson regression models: in the first model, the vaccine uptake was regressed on just the occupational group alone (bivariate analysis); in the second model, vaccine uptake was regressed on the occupational group alongside four other independent variables (adjusted covariates). ^b^ The IRR under multivariable analysis was adjusted for % Hispanic, % Black or African American, % female, and % less than 40 years old.

## Data Availability

The datasets used and/or analyzed in the current study are available upon reasonable request from the corresponding author.

## Supplementary Materials

The following supporting information can be downloaded at: https://www.mdpi.com/article/10.3390/vaccines10071000/s1, Table S1: The standard US occupational groups that were used for the current analysis.
