# Supplementary material for: COVID-19 Vaccine Uptake among US Adults According to Standard Occupational Groups
Source: Vaccines (Basel). 2022 Jun 23;10(7):1000. doi: 10.3390/vaccines10071000 (PMC9319436; doi:10.3390/vaccines10071000)
Supplement: Supplementary file 1 [file vaccines-10-01000-s001.zip › vaccines-1704104-supplementary.pdf]

Table S1. The standard US occupational groups that were used for the current analysis.

	Bivariate Analyses			Multivariable Analyses		
**Occupational Groups**	**IRR**	**95% CI**	**P-value**	**IRR**	**95% CI**	**P-value**
**Social Service/Protective Services**	0.99	0.97-0.99	<0.001	**0.996**	**0.988 - 1.005**	0.399
% Hispanic or Latino Population				0.995	0.994 - 0.996	**<0.001**
% Black or African American Population				0.995	0.994 - 0.996	**<0.001**
% Female				0.997	0.992 - 1.001	**<0.001**
% <40 years old				0.995	0.993 - 0.997	0.132
**Healthcare Practitioner/Technician****	1.03	1.02-1.03	<0.001	**1.008**	**1.003 - 1.014**	**0.001**
% Hispanic or Latino Population				0.996	0.995 - 0.997	**<0.001**
% Black or African American Population				0.995	0.994 - 0.996	**<0.001**
% Female				0.996	0.992 - 1.000	**0.074**
% <40 years old				0.995	0.993 - 0.997	**<0.001**
**Healthcare Support**	0.96	0.96-0.97	<0.001	**0.998**	**0.989 - 1.007**	0.693
% Hispanic or Latino Population				0.995	0.994 - 0.996	**<0.001**
% Black or African American Population				0.995	0.994 - 0.996	**<0.001**
% Female				0.996	0.992 - 1.001	0.121
% <40 years old				0.995	0.993 - 0.997	**<0.001**
**Business/Management/Legal**	1.02	1.01-1.02	<0.001	**1.011**	**1.008 - 1.013**	**<0.001**
% Hispanic or Latino Population				0.998	0.997 - 1.000	**0.009**
% Black or African American Population				0.997	0.996 - 0.998	**<0.001**
% Female				0.997	0.993 - 1.002	0.201
% <40 years old				0.996	0.994 - 0.998	**<0.001**
**Computer/Engineering/Life/Physical/Social Science**	1.03	1.02-1.03	<0.001	**1.018**	**1.013 - 1.023**	**<0.001**
% Hispanic or Latino Population				0.998	0.997 - 0.999	**<0.001**
% Black or African American Population				0.997	0.996 - 0.998	**<0.001**
% Female				0.997	0.992 - 1.001	0.152
% <40 years old				0.993	0.992 - 0.995	**<0.001**
**Arts/Design/Entertainment/Sports/Media**	1.08	1.06-1.09	<0.001	**1.031**	**1.018 - 1.044**	**<0.001**
% Hispanic or Latino Population				0.996	0.995 - 0.997	**<0.001**
% Black or African American Population				0.995	0.994 - 0.996	**<0.001**
% Female				0.997	0.993 - 1.001	0.172
% <40 years old				0.995	0.993 - 0.997	**<0.001**
**Office/Administrative Support**	0.99	0.98-0.99	<0.001	**0.997**	**0.993 - 1.001**	0.089
% Hispanic or Latino Population				0.995	0.994 - 0.996	**<0.001**
% Black or African American Population				0.995	0.994 - 0.996	**<0.001**
% Female				0.997	0.992 - 1.001	0.144
% <40 years old				0.995	0.993 - 0.997	**<0.001**
**Farming/Fishing/Forestry**	0.93	0.90-0.96	<0.001	**0.989**	**0.958 - 1.021**	0.485
% Hispanic or Latino Population				0.995	0.994 - 0.996	**<0.001**
% Black or African American Population				0.995	0.994 - 0.995	**<0.001**
% Female				0.996	0.992 - 1.001	0.112
% <40 years old				0.995	0.993 - 0.997	**<0.001**
**Building/Installation Maintenance Repair**	0.98	0.97-0.98	<0.001	**0.991**	**0.987 - 0.995**	**<0.001**
% Hispanic or Latino Population				0.997	0.996 - 0.998	**<0.001**
% Black or African American Population				0.995	0.994 - 0.996	**<0.001**
% Female				0.995	0.991 - 1.000	**0.041**
% <40 years old				0.995	0.993 - 0.997	**<0.001**
**Construction/Extraction/Production**	0.99	0.98-0.99	<0.001	**0.991**	**0.988 - 0.995**	**<0.001**
% Hispanic or Latino Population				0.998	0.997 - 0.999	**0.004**
% Black or African American Population				0.995	0.994 - 0.996	**<0.001**
% Female				0.996	0.992 - 1.001	0.115
% <40 years old				0.995	0.993 - 0.997	**<0.001**
**Transportation/Material Moving**	0.97	0.97-0.98	<0.001	**0.992**	**0.987 - 0.997**	**0.002**
% Hispanic or Latino Population				0.996	0.995 - 0.997	**<0.001**

% Black or African American Population				0.995	0.994 - 0.996	**<0.001**
% Female				0.996	0.992 - 1.001	0.095
% <40 years old				0.995	0.993 - 0.997	**<0.001**
**Food Preparation/Serving Related**	0.98	0.97-0.98	<0.001	**0.995**	**0.990 - 0.999**	**0.023**
% Hispanic or Latino Population				0.995	0.995 - 0.996	**<0.001**
% Black or African American Population				0.995	0.994 - 0.996	**<0.001**
% Female				0.996	0.992 - 1.000	0.078
% <40 years old				0.995	0.994 - 0.997	**<0.001**
**Personal Care/Service**	0.98	0.97-0.98	<0.001	**0.991**	**0.985 - 0.998**	**0.017**
% Hispanic or Latino Population				0.995	0.994 - 0.996	**<0.001**
% Black or African American Population				0.995	0.994 - 0.996	**<0.001**
% Female				0.997	0.992 - 1.001	0.129
% <40 years old				0.995	0.993 - 0.997	**<0.001**
**Sales and Sales Related**	1.01	1.01-0.02	<0.001	**0.999**	**0.994 - 1.004**	0.658
% Hispanic or Latino Population				0.995	0.994 - 0.996	**<0.001**
% Black or African American Population				0.995	0.994 - 0.995	**<0.001**
% Female				0.996	0.992 - 1.001	0.120
% <40 years old				0.995	0.993 - 0.997	**<0.001**
**Education/Training/Library**	1.03	1.02-1.04	<0.001	**1.002**	**0.995 - 1.008**	0.628
% Hispanic or Latino Population				0.995	0.994 - 0.996	**<0.001**
% Black or African American Population				0.995	0.994 - 0.996	**<0.001**
% Female				0.996	0.992 - 1.001	0.108
% <40 years old				0.995	0.993 - 0.997	**<0.001**
